# Assessment of Abuse Potential of Three Indazole-Carboxamide Synthetic Cannabinoids 5F-ADB, MDMB-4en-PINACA and ADB-4en-PINACA

**DOI:** 10.3390/ijms26136409

**Published:** 2025-07-03

**Authors:** Yanling Qiao, Xuesong Shi, Kaixi Li, Lixin Kuai, Xiangyu Li, Bin Di, Peng Xu

**Affiliations:** 1 School of Pharmacy, China Pharmaceutical University, Nanjing 210009, China; qiaoyanling1@126.com (Y.Q.); shixuesong0109@yeah.net (X.S.); cathyli0209@163.com (K.L.); kuailx@163.com (L.K.); 2 Office of China National Narcotics Control Commission, China Pharmaceutical University Joint Laboratory on Key Technologies of Narcotics Control, Beijing 100193, China; lixiangyu_ft@yeah.net; 3 Key Laboratory of Drug Monitoring and Control, Drug Intelligence and Forensic Center, Ministry of Public Security, Beijing 100193, China

**Keywords:** synthetic cannabinoids, tetrad effects, conditioned place preference, precipitated withdrawal, abuse potential

## Abstract

5F-ADB, MDMB-4en-PINACA and ADB-4en-PINACA are three potent indazole-carboxamide synthetic cannabinoids (SCs) that have been widely abused in recent years. However, the pharmacological research on these compounds remains limited, especially in vivo research data. The purpose of the present study was to investigate the pharmacological effects of 5F-ADB, MDMB-4en-PINACA and ADB-4en-PINACA in mice, comparing their in vivo effects with those caused by Δ^9^-tetrahydrocannabinol (Δ^9^-THC), the main psychoactive substance in cannabis. We evaluated the cannabinoid-specific pharmacological effects of 5F-ADB, MDMB-4en-PINACA and ADB-4en-PINACA using the tetrad assay (locomotion inhibition, hypothermia, analgesia and catalepsy). Then we conducted conditioned place preference (CPP) and precipitated withdrawal assay to assess the rewarding effect and physical dependence, with Δ^9^-THC as a positive control. The results showed that all of the three SCs exhibited potential tetrad effects in a dose-dependent manner, with median effective dose (ED_50_) values ranging from 0.03 to 0.77 mg/kg. In the CPP tests, they all exhibited a significant biphasic effect of conditioned place preference (CPP) and conditioned place aversion (CPA). A significant increase in paw tremors and head twitches was observed in the rimonabant-precipitated withdrawal assay, indicating that the repeated administration of these SCs can lead to potential physical dependence. All effective doses were lower than Δ^9^-THC. These findings strongly suggested that the three SCs exhibited similar but stronger cannabinoid-specific tetrad effects, rewarding effect and physical dependence compared with Δ^9^-THC, indicating their high abuse potential and possible threats to human health. The rank order of abuse potential for these drugs was 5F-ADB > MDMB-4en-PINACA > ADB-4en-PINACA > Δ^9^-THC.

## 1. Introduction

**Figure 1:**
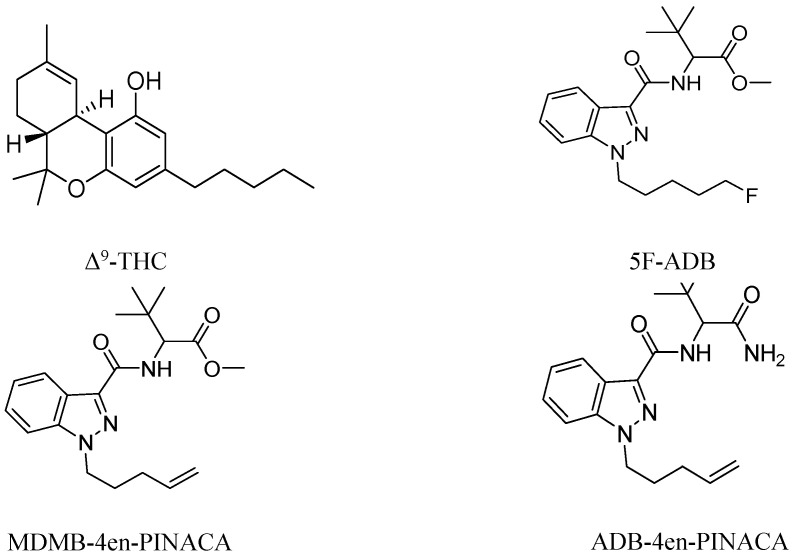
Chemical structures of Δ^9^-THC, 5F-ADB, MDMB-4en-PINACA and ADB-4en-PINACA evaluated in the present study.

Synthetic cannabinoids (SCs) are novel psychoactive substances that first appeared as a legal alternative to cannabis on the illicit drug market around the year 2000. Despite a global decline in SC abuse, SCs still account for about one-third of all novel psychoactive substances appearing on the global market [1]. The abuse of SCs poses a serious threat to public health and safety. These compounds are generally more potent than natural cannabinoids and tend to produce a stronger cannabimimetic effect than phytocannabinoid Δ^9^-tetrahydrocannabinol (Δ^9^-THC, Figure 1), the primary psychoactive component of cannabis [2]. Compared with cannabis, severe and fatal poisoning appears to be more common under the influence of SCs. They are known to produce a variety of clinical symptoms including cardiotoxicity, respiratory depression, renal injury, cognitive disruption, motor disorders, nausea, vomiting, dizziness, headache, coma, epilepsy, anxiety, paranoia, hallucinations, mental disorders etc., and have been linked to numerous fatalities [3,4,5,6,7,8,9]. SCs exert their effects by binding to and activating endogenous G protein-coupled cannabinoid receptors (CB1 and CB2), similar to Δ^9^-THC, with their psychoactive effects primarily mediated by CB_1_ receptor (CB_1_R) interactions [1].

In recent years, indazole-carboxamide-class SCs have emerged and become more prevalent. The in vitro and in vivo pharmacology of many of these SCs has been explored, but the animal behavior experimental data on addiction and toxicity of most newer analogs remain limited. 5F-ADB, MDMB-4en-PINACA and ADB-4en-PINACA (Figure 1) are three analogs with the same indazole cores.

5F-ADB, also known as 5F-MDMB-PINACA or methyl 2-[1-(5-fluoropentyl)-1H-indazole-3-carbonyl] amino-3,3-dimethylbutanoate, has been sold on the drug market since at least the end of 2014. It is one of the most potent and dangerous indazole-based synthetic cannabinoids. Although the mechanism of toxicity remains unclear, it has been implicated in many cases of non-fatal intoxication and fatalities reported worldwide [10,11,12]. The research on 5F-ADB has mainly focused on the identification and analysis methods as well as metabolism, and some pharmacological studies have also been reported. 5F-ADB bound to CB1R with high affinity K_i_ = 0.42 nM, which is 81 times stronger than Δ^9^-THC (K_i_ = 34 nM), has been studied [13]. Banister et al. found that 5F-ADB acted as a potent, highly efficacious agonist at CB1R (half-maximal effective concentration, EC_50_ = 0.59 nM, 290 times more potent than Δ^9^-THC) and CB2R (EC_50_ = 7.5 nM) in a fluorometric membrane potential assay [14]. Antonides et al. investigated the activation of 5F-ADB with CB1R and CB2R using a G protein-coupled receptor (GPCR) activation assay based on split NanoLuc luciferase complementation, reporting an EC_50_ of 1.78 nM and 1.46nM, respectively. It demonstrated a higher potency at the CB2R than at the CB1R [15,16]. Interestingly, several phase I metabolites of 5F-ADB retain affinity, act as high-efficacy agonists and exhibit atypical pharmacodynamic properties at CB1R. They may accumulate with the parent drug to produce severe toxicity [13]. In vivo studies found that 5F-ADB produced short-acting depression of locomotor activity with median effective dose (ED_50_) of 1.1 mg/kg and fully substituted for the discriminative stimulus effects of Δ^9^-THC in mice with ED_50_ = 0.07 mg/kg [17]. A study by Asaoka et al. suggested that 5F-ADB activates the local CB1R and potentiates midbrain dopaminergic systems with no direct effects on midbrain serotonergic systems [18].

MDMB-4en-PINACA [methyl-3,3-dimethyl-2-(1-(pent-4-en-1-yl)-*1H*-indazole-3-carboxamido) butanoate)], also called MDMB-PENINACA, or ADB-PINACA-A, shares structural similarities with 5F-ADB. It differs from 5F-ADB due to the replacement of 5-fluoropentyl (5F) with a pent-4-ene (4-en) group [19]. It has been available on the European drug market since at least 2017 and its illicit use was first reported by Slovenia in 2018 [20,21]. It quickly became one of the most prevalent SCs and one of the most commonly detected SCs in prisons [3]. MDMB-4en-PINACA is a full and potent synthetic cannabinoid receptor agonist (SCRA). It has significant physiological effects, is associated with a wide range of adverse effects and may cause or contribute to death [22]. In vitro studies have shown its high potency and efficacy at CB1R with an EC_50_ of 0.680–3.30 nM based on βarr2 assay [19,22,23,24], mini-Gα_i_ assay [25], [^35^S]-GTP_γ_S assay [25,26] or AequoZen cell assay system [27], with an E_max_ of 106–299% (compared with JWH-018) or E_max_ of 654% (compared with Δ^9^-THC). Different data observed between the βarr2 and mini-Gαi assays were the best predictor for ‘biased agonism’ towards βarr2 or G protein recruitment [23]. MDMB-4en-PINACA exhibited a 3-fold greater potency at the hCB2 receptor (EC_50_ = 1.34 nM) than the hCB1 receptor (EC_50_ = 3.30 nM), and exhibited a 27-fold greater potency at the hCB1 receptor than Δ^9^-THC (EC_50_ = 89.9 nM) and a 15-fold greater potency at the hCB2 receptor than Δ^9^-THC (EC_50_ = 20.3 nM) [26]. Meanwhile, its binding affinity K_i_ values at the hCB1 and the hCB2 receptor were 1.4 nM and 0.213 nM, respectively. It exhibited a 7-fold greater affinity at the hCB_2_ receptor than the hCB_1_ receptor, and exhibited a 6-fold greater affinity at the hCB1 receptor than Δ^9^-THC (K_i_ = 8.05 nM)and a 150-fold greater affinity at the hCB2 receptor than Δ^9^-THC (K_i_ = 32 nM) [26]. A slightly lower binding affinity K_i_ = 0.28 nM was obtained in another study, which was 10 times higher than that of JWH-018 (K_i_ = 2.6 nM, approximately) [28]. According to in vivo research, MDMB-4en-PINACA produced hypothermia with shorter latency to peak effects than Δ^9^-THC and showed full, dose-dependent substitution for Δ^9^-THC in drug discrimination with ED_50_ = 0.025 mg/kg in male mice and ED_50_ = 0.051 mg/kg in female mice [26]. MDMB-4en-PINACA disrupts hippocampal function and impairs cognitive performance, highlighting the cognitive risk associated with SC abuse [29]. In zebrafish models, MDMB-4en PINACA exhibits dose-dependent behavioral effects: low doses produce anxiolytic effects accompanied by increased aggression, while high doses induce hallucinogenic responses, potentially mediated through the modulation of neurotransmitter systems including serotonin, dopamine, acetylcholine and histamine [30]. It could induce hypolocomotion, hypothermia and analgesia in mice even at a low dose of 0.1 mg/kg [31]. The dose-dependent behavioral changes in zebrafish and the cannabinoid-specific physiological responses in mice collectively demonstrate MDMB-4en-PINACA’s potent agonist activity at the CB1 receptors in vivo. These effects are consistent with its high binding affinity and functional potency at the CB1 receptors observed in vitro, reinforcing its role as a full and efficacious SCRA.

ADB-4en-PINACA, also known as ADMB-4en-PINACA, ADB-PENINACA and (N-(1-carbamoyl-2,2-dimethyl-propyl)-1-pent-4-enyl-indazole-3-carboxamide), was first detected in plant-like drug material in the United States in 2021. Recently, ADB-4en-PINACA and MDMB-4en-PNACA were found to be adulterants in tianeptine, an atypical antidepressant in United States. This contamination may explain the sudden rise in reported adverse events, which significantly exceeded the expected annual background incidence rate for tianeptine alone [32]. Existing studies have detailed the synthesis [27], detection methods, metabolism [27,33], pharmacokinetics [31] and in vitro pharmacological evaluation of ADB-4en-PINACA [23,25,28]. ADB-4en-PINACA demonstrated a higher binding affinity at human CB1 receptors (K_i_ = 0.17 nM) compared with MDMB-4en-PINACA (K_i_ = 0.28 nM) [28]. However, functional assays revealed lower in vitro potency, with EC_50_ values of 3.43 nM (βarr2 assay), 1.45 nM (mini-Gα_i_ assay) and 1.58 nM ([^35^S]-GTP_γ_S assay), compared with MDMB-4en-PINACA (1.88 nM, 0.993 nM and 0.680 nM, respectively) [25]. Another in vitro result also showed that ADB-4en-PINACA (EC_50_ = 11.6 nM) was less potent than MDMB-4en-PINACA (EC_50_ = 4.3 nM) and more potent than JWH-018 (EC_50_ = 28.2 nM) [27]. Its cannabimimetic effects have been reported recently [31].

Although in vitro binding affinity, potency and efficacy at cannabinoid receptors of the three SCs have been reported, the systematic evaluation in vivo, especially regarding psychological and physical dependence potential, remains insufficiently explored for now. In this study, we comprehensively assessed the pharmacological effects of 5F-ADB, MDMB-4en-PINACA and ADB-4en-PINACA using tetrad experiments, conditioned place preference (CPP) experiments and precipitated withdrawal experiments, with Δ^9^-THC as a positive control, and our findings demonstrated the significant abuse potential of the three indazole-carboxamide synthetic cannabinoids.

## 2. Results

### 2.1. Three SCs Induced Tetrad Effects

**Figure 2:**
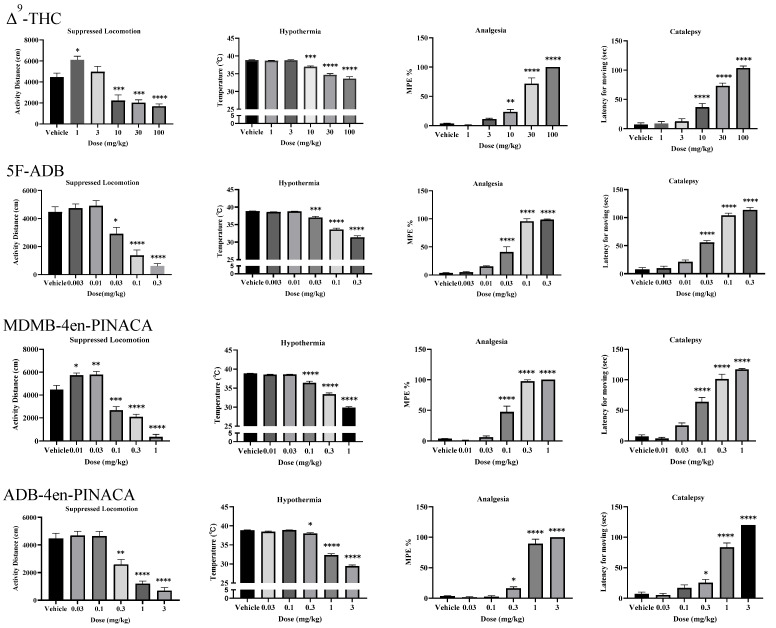
Tetrad effects of Δ^9^-THC (1–100 mg/kg, i.p.), 5F-ADB (0.003–0.3 mg/kg, i.p.), MDMB-4en-PINACA (0.01–1 mg/kg, i.p.) and ADB-4en-PINACA (0.03–3 mg/kg, i.p.) in mice. Each column represented mean ± SEM of data from 8 to 12 mice. * *p* < 0.05; ** *p* < 0.01; *** *p* < 0.001; **** *p* < 0.0001 compared with the vehicle group.

Locomotor activity, body temperature, analgesia and catalepsy effects were observed 30 min after the intraperitoneal injection of the three SCs and Δ^9^-THC. All drugs exhibited characteristic dose-dependent tetrad effects (Figure 2) and the ED_50_ values are shown in Table 1.

Δ9-THC at 1 mg/kg significantly increased locomotor activity in the open field test, but higher doses (10–100 mg/kg) significantly suppressed locomotion [F(5,52) = 24.16, *p* < 0.05], decreased body temperature [F(5,64) = 60.75, *p* < 0.05], increased pain stimulation threshold in the tail flick test [F(5,57) = 101.2, *p* < 0.05] and induced catalepsy in the horizontal bar test [F(5,54) = 91.10, *p* < 0.05].

5F-ADB at 0.003–0.01 mg/kg did not significantly affect locomotor activity compared with the control group. When the dose was 0.03–0.3 mg/kg, 5F-ADB significantly inhibited locomotor activity [F(5,48) = 23.22, *p* < 0.05], decreased body temperature [F(5,58) = 113.2, *p* < 0.05], increased tail flick latency [F(5,60) = 158.4, *p* < 0.05] and induced catalepsy in the horizontal bar test [F(5,66) = 168.9, *p* < 0.05] significantly.

MDMB-4en-PINACA increased locomotion at 0.01–0.03 mg/kg but caused significant suppression at 0.1–1 mg/kg [F(5,60) = 64.02, *p* < 0.05]. The 0.1–1 mg/kg dose also induced hypothermia [F(5,56) = 227.2, *p* < 0.05], analgesia [F(5,59) = 122.6, *p* < 0.05] and catalepsy [F(5,61) = 91.04, *p* < 0.05].

ADB-4en-PINACA did not significantly affect locomotion at 0.03–0.1 mg/kg, but caused significant suppression at 0.3–3 mg/kg [F(5,55) = 31.45, *p* < 0.05]. The 0.3–3 mg/kg dose also induced hypothermia [F(5,66) = 354.1, *p* < 0.05], analgesia [F(5,58) = 203.8, *p* < 0.05], and catalepsy [F(5,63) = 116.8, *p* < 0.05].

### 2.2. Drug-Induced CPP

**Figure 3:**
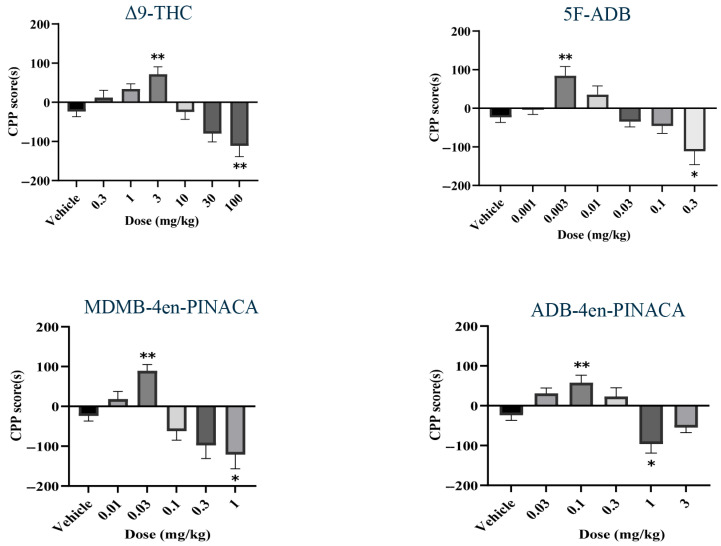
Conditioned place preference (CPP) scores (difference between the time spent in the drug-paired and in the nondrug-paired compartments) for Δ^9^-THC, 5F-ADB, MDMB-4en-PINACA and ADB-4en-PINACA during the post-conditioning test session. Each column represents mean ± SEM of data from 8 to 12 mice. * *p* < 0.05; ** *p* < 0.01 compared with the vehicle group.

The rewarding effects of Δ^9^-THC, 5F-ADB, MDMB-4en-PINACA and ADB-4en-PINACA were evaluated using the CPP paradigm. As shown in Figure 3, all three SCs and Δ^9^-THC induced CPP effects at low doses and CPA effects at high doses, exhibiting a biphasic, dose-dependent response (one-way ANOVA, Δ^9^-THC: F(6,68) = 11.16, *p* < 0.05; 5F-ADB: F(6,73) = 8.651, *p* < 0.05; MDMB-4en-PINACA: F(5,61) = 10.61, *p* < 0.05; ADB-4en-PINACA: F(5,52) = 10.22, *p* < 0.05). Δ^9^-THC at the dose of 3 mg/kg increased the time spent in the drug-paired compartment, while higher doses (100 mg/kg) produced place aversion. Similarly, 5F-ADB (0.003 mg/kg), MDMB-4en-PINACA (0.03 mg/kg) and ADB-4en-PINACA (0.1 mg/kg) significantly increased drug-paired compartment time, with place aversion observed at higher doses (0.3 mg/kg, 1 mg/kg and 1 mg/kg, respectively). The CPP scores followed an inverted U-shaped dose–response pattern.

### 2.3. Evaluation of Drug Withdrawal

**Figure 4:**
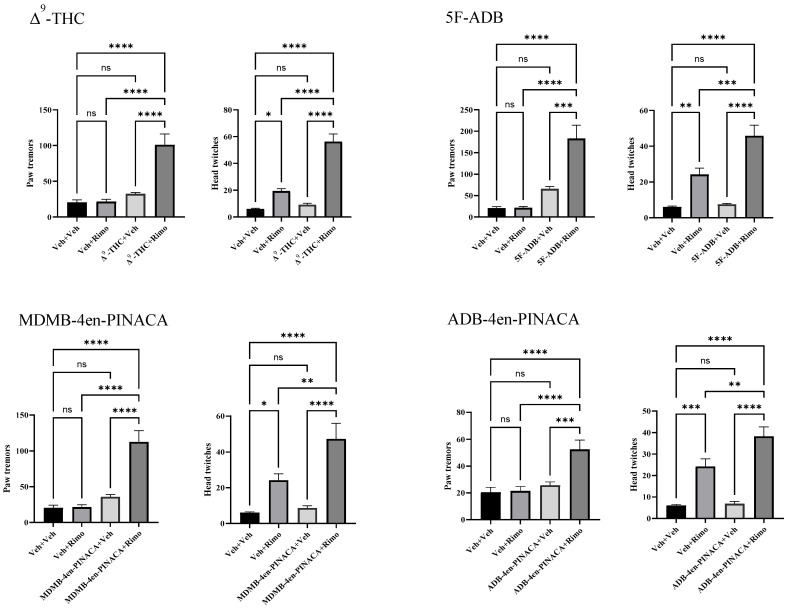
Mice were treated with Δ9-THC (increasing doses of 1, 3, 10, 30, 100 mg/kg, i.p.), 5F-ADB (increasing doses of 0.003, 0.01, 0.03, 0.1, 0.3 mg/kg, i.p.), MDMB-4en-PINACA (increasing doses of 0.01, 0.03, 0.1, 0.3, 1 mg/kg, i.p.) and ADB-4en-PINACA (increasing doses of 0.03, 0.1, 0.3, 1, 3 mg/kg, i.p.) twice daily for 5 consecutive days to induce addiction. Rimonabant (3 mg/kg, i.p.) was administered on day 6 to precipitate withdrawal. Data are expressed as mean ± SEM, n = 8–12 of each group. * *p* < 0.05; ** *p* < 0.01; *** *p* < 0.001; **** *p* < 0.0001.

The physical dependence of 5F-ADB, MDMB-4en-PINACA, ADB-4en-PINACA and Δ^9^-THC was evaluated through precipitated withdrawal assays (Figure 4). Acute rimonabant administration (3 mg/kg, i.p.) precipitated physical withdrawal symptoms in mice after chronic treatment with Δ^9^-THC (1–100 mg/kg), 5F-ADB (0.003–0.3 mg/kg), MDMB-4en-PINACA (0.01–1 mg/kg) and ADB-4en-PINACA (0.03–3 mg/kg). Drug treatment alone (drug + veh groups) did not induce significant withdrawal symptoms compared with the vehicle controls (paw tremors: all *p* > 0.05; head twitches: all *p* > 0.05).

All drug-treated groups showed significant increases in withdrawal symptoms compared with the vehicle controls. For paw tremors, one-way ANOVA showed a significant effect of drug treatments (Δ^9^-THC F(3,39) = 19.41, *p* < 0.05; 5F-ADB F(3,41) = 20.43, *p* < 0.05; MDMB-4en-PINACA F(3,41) = 24.90, *p* < 0.05; ADB-4en-PINACA F(3,41) = 11.40, *p* < 0.05). The mean paw tremors counts were 100.9, 182.8, 112.6 and 52.5 for Δ9-THC, 5F-ADB, MDMB-4en-PINACA and ADB-4en-PINACA, respectively, which were all significantly higher than the vehicle controls. Similarly, head twitches were significantly increased (Δ^9^-THC F(3,43) = 52.91, *p* < 0.05; 5F-ADB F(3,39) = 29.29, *p* < 0.05; MDMB-4en-PINACA F(3,39) = 19.76, *p* < 0.05; ADB-4en-PINACA F(3,41) = 29.30, *p* < 0.05). The mean counts for head twitches for Δ^9^-THC, 5F-ADB, MDMB-4en-PINACA and ADB-4en-PINACA were 56.3, 45.8, 47.3 and 38.3, respectively. The acute administration of rimonabant induced head twitch symptoms in mice after the chronic administration of the vehicle, but the response was weaker than the drug treatment groups.

## 3. Discussion

The cannabinoid-induced tetrad is a preclinical model to test whether compounds produce cannabinoid-like behavioral effects, suggesting that they might be CB1 agonists. However, to definitively establish CB1 receptor mediation, these behavioral findings must be corroborated with binding assays and functional assays. This model assesses four specific phenotypic effects of CB1 agonists: hypolocomotion, hypothermia, analgesia and catalepsy [34]. In this study, we slightly modified the classical cannabinoid tetrad assay, originally developed by B.R. Martin [35] to evaluate three indazole-carboxamide SCs, namely 5F-ADB, MDMB-4en-PINACA and ADB-4en-PINACA with Δ^9^-THC as a positive control [34]. The results showed that all three SCs induced the typical tetrad effects. They significantly decreased locomotion, body temperature and pain sensitivity, and impaired the capacity to initiate movement in mice. The potency ranking was 5F-ADB > MDMB-4en-PINACA > ADB-4en-PINACA > Δ^9^-THC, with approximately a 375-, 107- and 25-fold higher potency than Δ^9^-THC, respectively, which is consistent with previous reports [14,26,27]. The results highlight the unpredictable effects and potential for severe adverse reactions. Notably, 5F-ADB was the most potent, likely due to its 5-fluoropentyl tail enhancing blood–brain barrier penetration and CB1 binding affinity, as supported by prior work on fluorinated psychoactives [36].

Motor impairment is one of the main behavioral effects observed after the systemic administration of cannabinoid receptor agonists, posing a significant threat to physical health and public safety [37]. Our study demonstrated that MDMB-4en-PINACA (0.01 mg/kg and 0.03 mg/kg) and Δ^9^-THC (1 mg/kg) significantly increased the locomotor activity, and was significantly inhibited as the doses increased, which demonstrated the biphasic modulation effect of locomotor activity in mice. We confirmed the previous findings of the dose-dependent motor stimulating and inhibiting effects of the cannabinoids receptor agonist [9,38]. But, this result was inconsistent with the recent literature which found that the administration of MDMB-4en-PINACA elicited an immediate hypolocomotive response [31]. The data are also inconsistent with earlier reports showing that 5F-ADB produced short-acting locomotor depression (ED_50_ = 1.1 mg/kg) without prior stimulation [17]. This discrepancy may reflect differences in dosing regimens or animal models.

We observed notable differences in the relative potency of tetrad effects across the tested compounds. Δ9-THC and ADB-4en-PINACA were 3–5 times and 2–3 times more potent in suppressing locomotion than in producing hypothermia, analgesia or catalepsy. In contrast, the potencies of hypothermia induced by MDMB-4en-PINACA and 5F-ADB were 3–4 times and 2 times lower, respectively, than those of locomotion suppression, analgesia and catalepsy. 5F-ADB exhibited the lowest hypothermia potency among all tetrad effects. This might be related to differences in the key brain regions and mechanisms of tetrad effects induced by these drugs, as well as differences in chemical structures. For instance, subtle variations in side chain modifications or core scaffolds may alter receptor binding kinetics or regional brain distribution, leading to the selective amplification or dampening of specific effects. Further studies mapping CNS activation patterns could clarify these mechanistic distinctions.

Case reports have outlined that the three SCs may possess high abuse potential [21,32,39], but there is rare direct evidence supporting this speculation, especially in vivo data. In the present study, we systematically evaluated the abuse potential of the three SCs using both the CPP paradigm and the precipitated withdrawal paradigm.

Place conditioning is a classic assay to evaluate the rewarding effect in vivo, which has revealed the psychological dependence of drugs. Many compounds such as NM-2201, SDB-005 and Δ^9^-THC have shown a biphasic effect, which could induce CPP at low doses and induce CPA at high doses [40,41,42,43]. In the present study, we concluded that the three SCs and Δ^9^-THC all possessed a rewarding effect and aversive effect that induced significant CPP and CPA in a dose-dependent manner by intraperitoneal injection. This phenomenon may relate to the release of dopamine in the nucleus accumbens shell (NAc shell), as demonstrated by the inverted U-shaped DA release patterns following SC administration [44]. However, the exact CPA mechanisms remain unclear, as high-dose aversion could represent either true drug aversion or motor impairment—a distinction requiring future studies with operant assays or microdialysis during conditioning. In contrast, higher doses that induce CPA could lead to nonspecific locomotor suppression (as seen in our tetrad data). Notably, the minimal effective CPP doses were substantially lower than those required for locomotor suppression: 5F-ADB induced CPP at a dose of 0.003 mg/kg, which is 1/10 of the dose which suppressed locomotion significantly. MDMB-4en-PINACA and ADB-4en-PINACA induced CPP at a dose of 0.03 mg/kg and 0.1 mg/kg, respectively, which is 1/3 of the dose which could suppress locomotion significantly. Compared with the dose of Δ^9^-THC used to induce CPP (3 mg/kg), 5F-ADB showed the highest potency to induce CPP, followed by MDMB-4en-PINACA and ADB-4en-PINACA. It indicated that the rewarding effects of 5F-ADB, MDMB-4en-PINACA and ADB-4en-PINACA were approximately 1000 times, 100 times and 30 times that of Δ^9^-THC, respectively. Although the potency ratios may differ from in vitro data reported previously, in which the EC_50_s were approximately 290 times (5F-ADB), 27 times (MDMB-4en-PINACA) and 10 times (ADB-4en-PINACA) that of Δ9-THC, respectively, the rank order was consistent [14,26,27]. The differences in the ratios also suggested that the potency obtained from in vitro experiments sometimes cannot fully reflect the in vivo effects of drugs. Meanwhile, we can speculate that the three SCs all have high abuse potential. However, in our study, Δ^9^-THC elicited CPP at 3 mg/kg and CPA at 100 mg/kg, which is higher than that reported before, where CPP doses were approximately 0.1–0.3 mg/kg and CPA doses were 1 mg/kg and above [45]. This may be related to the different types of experimental animals used and different administration method. It is worth noting that the observed CPA may be due to either a drug aversion effect or a locomotor suppression effect (as demonstrated in our tetrad assay (Figure 2)). The reasons for this phenomenon need further research. Despite the relatively modest CPP magnitudes (~100 s), the statistical significance of low-dose preference, combined with the dose-dependent shift to aversion, supports the biological relevance of these findings. While stronger effects (200–300 s) are typical for highly reinforcing drugs (e.g., opioids), cannabinoids often produce subtler behavioral responses, as seen in prior studies [46,47]. The inverted U-shaped dose–response curve, coupled with the dissociation between rewarding and locomotor-suppressive doses, underscores the high abuse potential of these SCs. Future studies could refine the conditioning protocols to enhance the sensitivity to cannabinoid-specific reward phenotypes.

Withdrawal signs were induced by the synthetic cannabinoids and the physical withdrawal syndrome closely resembled that seen in cannabis dependence [48]. The CB1 receptor antagonist rimonabant precipitates cannabinoid withdrawal symptoms in rodents mainly including head twitches, face rubbing, scratching, wet dog shakes, piloerection and front paw tremors [49,50,51]. In our withdrawal assay, we selected two common and easily observable signs—paw tremors and head twitches—as the primary withdrawal measures for the experiment. The results showed that repeated administration for 5 days in a dose-increasing manner, 5F-ADB, MDMB-4en-PINACA and ADB-4en-PINACA could induce paw tremors and head twitches in mice after injecting rimonabant (Figure 4). Compared with the negative group, 5F-ADB caused the most paw tremors, followed by MDMB-4en-PINACA and ADB-4en-PINACA. For head twitches, 5F-ADB and MDMB-4en-PINACA showed comparable frequencies, both more than that of ADB-4en-PINACA. Overall, 5F-ADB caused the strongest physical withdrawal symptoms, while ADB-4en-PINACA was the weakest one. Meanwhile, the initial dose of 5F-ADB in the withdrawal experiment is lower than that of MDMB-4en-PINACA, ADB-4en-PINACA and Δ^9^-THC. These results suggest that the physical dependence potency ranking was 5F-ADB > MDMB-4en-PINACA > ADB-4en-PINACA > Δ^9^-THC, like in the tetrad. It should be pointed out that rimonabant alone also caused head twitches, as seen in the vehicle–rimonabant group (Veh + Rim). This phenomenon has been reported in the literature [52]. As a potent CB1 receptor antagonist, rimonabant can precipitate mild withdrawal-like symptoms even in drug-naive animals by blocking basal endocannabinoid tone. Importantly, our data demonstrated that the Drug + Rim groups showed greater symptom frequencies than the Veh + Rim controls (*p* < 0.05). This phenomenon suggested that, while rimonabant alone produced baseline effects, the amplified responses in SC-pretreated animals specifically reflect cannabinoid dependence. The Veh + Rim data serve as a crucial baseline for distinguishing pharmacological withdrawal from nonspecific effects.

Understanding the structure–activity relationship (SAR) is crucial for predicting the pharmacological effects of new analogs, evaluating the abuse potential of structural analogs and providing important reference information for drug control departments. In this study, we sought to elucidate the structure-activity relationships according to the results of in vivo experiments. The three SCs tested in this study all belong to the indazole-carboxamide family. They share the same indazole “core”, carboxamide “neck” (linker) and differences in the “head” and “tail” groups. Based on the analysis of the present results, we concluded that modifications to the head and tail regions led to some differences in potency. The name of 5F-ADB (5F-MDMB-PINACA) is derived from its structural features: a fluoro moiety at position 5 of the pentyl chain (5F), a dimethyl methyl butanoate (tert-leucinate) head (MDMB), a pentyl tail (P), an indazole core (INA) and a carboxamide linker (CA). Structurally, MDMB-4en-PINACA contains a pent-4-ene moiety on a pentyl tail, i.e., it differs in the tail (5-fluoropentyl) from 5F-ADB. Halogen substitution enhances lipophilicity, facilitating blood–brain barrier penetration. Canazza et al. demonstrated that fluorination can increase the power and/or effectiveness of SCs [53]. The present study showed that 5F-ADB is more potent in inducing tetrad effects, CPP and precipitation of withdrawal compared with MDMB-4en-PINACA, probably due to its fluorination, which may contribute to the rapid penetration across the blood–brain barrier into the central nervous system. Gillis et al. [54] reported that fluorination is widely recognized to improve both receptor binding affinity (2–5 fold potentiation) and metabolic stability in psychoactive compounds, supporting our observation that 5F-ADB (average ED_50_ = 0.04 mg/kg) was 3.5 times more potent than MDMB-4en-PINACA (average ED_50_ = 0.14 mg/kg) in producing tetrad effects. In summary, the observed in vivo potencies reflect a combination of two key factors: (1) differential CB1 receptor binding affinities, as demonstrated in our cited in vitro studies [14,26,27], (2) compound-specific pharmacokinetic profiles, including blood–brain barrier penetration and metabolic stability. For instance, the superior potency of 5F-ADB likely stems from both its high CB1 affinity and enhanced brain bioavailability due to fluorination [53,54]. In addition, ADB-4en-PINACA has the same tail as MDMB-4en-PINACA, featuring an L-tert-leucinamide rather than a tert-leucinate “head”. In this study, it was observed that MDMB-4en-PINACA had stronger tetrad effects, rewarding effect and precipitation of withdrawal than ADB-4en-PINACA. We concluded that a tert-leucinate moiety in the “head” leads to a higher potency than the corresponding L-tert-leucinamide head group. The deduction was consistent with a previous study with the same structural analogs ADB-4en-PINACA and MDMB-4en-PINACA, in which the conclusion was reached that the presence of the L-tert-leucinate head group led to more potent and sustained effects than the L-tert-leucinamide head group [31]. These findings will help us study the structure–activity relationships of SCs from the perspective of animal experiments.

## 4. Materials and Methods

### 4.1. Animals

ICR mice (male, 18–22 g) were purchased from SPF Biotechnology Co., Ltd. (Beijing, China). The animals were housed in a humidity- (50 ± 10%) and temperature- (25 ± 2 °C) controlled environment on a 12 h dark/light cycle with water and food ad libitum. The animal use protocol listed below has been reviewed and approved by the Welfare and Ethics Committee for Laboratory Animals, Key Laboratory of Drug Monitoring and Control. Approval No. KLDMC-WECLA-202406-05.

### 4.2. Drugs

Δ^9^-THC, 5F-ADB, MDMB-4en-PINACA and ADB-4en-PINACA was provided by Drug Intelligence and Forensic Center, Ministry of Public Security (Beijing, China). Rimonabant hydrochloride was purchased from MedChemExpress (MCE, Monmouth Junction, NJ, USA). The drug was initially dissolved in absolute ethanol and diluted to the final working concentration with a vehicle mixture containing 5% (*v*/*v*) Tween 80 and normal saline before injection. Vehicle control solutions (ethanol + 5% Tween 80 + saline) were prepared in parallel.

### 4.3. Tetrad Assay

**Figure 5:**
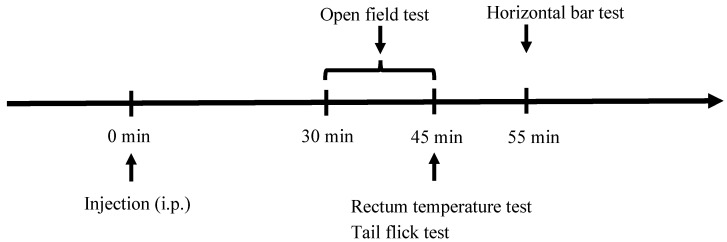
Experimental procedure of the tetrad assay.

The tetrad assay was performed to investigate whether the administration of the drugs could induce tetrad effects including suppressed locomotor activity, hypothermia, analgesia and catalepsy. Mice were acclimated to the test room for at least 30 min before the tetrad assay began. Then, experiments were sequentially tested for locomotor activity, body temperature, analgesia and catalepsy 30 min after administration based on methods from the literature with some modifications [34,55]. The experimental procedure of the tetrad assay is illustrated in Figure 5.

#### 4.3.1. Locomotor Activity

An open field test was used to evaluate the suppressed locomotion effect. Mice were placed into the center of the open field apparatus (40 cm × 40 cm × 30 cm). A video tracking system Tracking Master V3.63 (Beijing zhongshidichuang Science and Technology Development Co., Ltd., Beijing, China) was used to automatically record and analyze the distance traveled by each mouse for 15 min. Between the test of each mouse, the open field was cleaned with a paper towel moistened with 20% ethanol.

#### 4.3.2. Body Temperature

The core body temperature was measured using a mice rectal thermometer probe (Temperature maintenance device, model ZS-T, Beijing zhongshidichuang Science and Technology Development Co., Ltd., Beijing, China). The probe lubricated with Vaseline was gently inserted into the mouse’s rectum by approximately 2 cm until the temperature stabilized for approximately 10 s, and the temperature was recorded. Between each mouse, the probe was cleaned with a paper towel moistened with 20% ethanol.

#### 4.3.3. Analgesia

Analgesia was assessed using the tail flick test. The body of the mouse was held gently in the investigator’s hand and the tail was allowed to hang freely. The distal part of the tail (approximately 3 cm) was dipped into a water bath maintained at 50 ± 0.5 °C, and the tail flick latencies were recorded with a stopwatch. A 30s cut off time was used to avoid tissue damage. Baseline pain threshold served as the baseline response latency, which was assessed before the administration of the drugs. Tail flick latency data were represented as a percentage of the maximum possible effect (%MPE) to reflect the analgesia effect, calculated using the following formula: %MPE = [(measured response latency − baseline response latency)/(30 s baseline response latency)] × 100%.

#### 4.3.4. Catalepsy

Catalepsy was measured using the horizontal bar test. A cylindrical horizontal bar (0.5 cm in diameter) was fixed in a single mouse plastic cage at an elevation of 3–4 cm above the bottom. The mouse was lifted by the tail and its forelimbs placed gently on the horizontal bar with its hind legs on the bottom of the cage. The total latency for the mouse to move one or both forepaws off the bar was recorded, with a cut off time of 2 min. Between each mouse, the cage was cleaned with a paper towel moistened with 20% ethanol.

### 4.4. CPP Paradigm

The CPP apparatus consisted of a CPP box (40 cm × 23 cm × 30 cm), a camera and a computer (Beijing zhongshidichuang Science and Technology Development Co., Ltd., Beijing, China). The CPP box was divided into three compartments by two removable partitions. The left and right compartments were of equal size (16 cm× 17 cm× 30 cm) and were connected by a central compartment (8 cm × 17 cm × 30 cm). The side compartments were distinguished by visual and tactile cues. The left compartment had black walls with round holes on the floor, while the right compartment had black and white vertical stripes on the walls with dot and strip holes on the floor. The camera was suspended overhead to record the mice movements. The time spent in each compartment by the mice was automatically recorded and analyzed using Tracking Master V3.63 software.

The CPP procedure included three phases: pre-conditioning test, drug conditioning and post-conditioning test. Mice were allowed to freely explore all three compartments (partitions removed) for 15 min to acclimate to the apparatus the day before the pre-conditioning test.

#### 4.4.1. The Pre-Conditioning Test (Day 1)

On day 1, each mouse was placed in the central compartment with the two partitions removed, so that the mouse could move freely among the three compartments for 15 min. The residence time in each compartment was recorded. Each mouse was screened once in the morning and once in the afternoon. Mice with large differences in natural place preference between the left and right compartments were excluded from further study. The qualified mice whose difference in residence time between the left and right compartments during the two times was ≤150 s were taken for testing. They were randomly divided into several groups (n = 8–12 mice for each administration dose): vehicle control group (vehicle), Δ^9^-THC groups (0.3, 1, 3, 10, 30, and 100 mg/kg), 5F-ADB groups (0.001, 0.003, 0.01, 0.03, 0.1, and 0.3 mg/kg), MDMB-4en-PINACA groups (0.01, 0.03, 0.1, 0.3, and 1 mg/kg) and ADB-4en-PINACA groups (0.03, 0.1, 0.3, 1, and 3 mg/kg).

#### 4.4.2. Drug Conditioning (Days 2–11)

On days 2–11, the mice were trained for one session every day. Mice received an intraperitoneal injection of drugs or vehicle and were immediately placed in its nonpreferred compartment (drug-paired compartment) for 45 min with the channels inside the compartments closed with the partitions. The next day, the same mouse was injected with vehicle and immediately placed in its preferred compartment (nondrug-paired compartment) for 45 min. A total of five cycles were conducted, with two days counted as one cycle.

#### 4.4.3. The Post-Conditioning Test (Day 12)

Twenty-four hours after the final vehicle administration, the mice were placed in the middle compartment without injection and allowed to explore the compartments freely for 15 min by removing the two partitions. The time spent in each compartment was recorded. The CPP score of each group was calculated by the time spent in the drug-paired compartment minus the time spent in the nondrug-paired compartment on the test day, which served as an indicator of drug-induced place preference.

### 4.5. Precipitated Withdrawal Assay

The mice were randomly divided into several groups (n = 8–12 mice for each administration dose). Each group was administered drugs or vehicle twice a day (at 9:00 and 19:00) for 5 consecutive days. The initial doses were selected based on the tetrad assay and the doses increased threefold every day (showed in Table 2). On the morning of day 6, all mice received a final injection of drugs, where the dose administered was the same as on day 5. Four hours later, mice received an intraperitoneal injection of CB1R antagonist rimonabant (3 mg/kg) [51] to precipitate withdrawal. The vehicle control group received a vehicle injection.

Immediately after rimonabant injection, the mice were placed individually in a transparent observation cylinder (diameter 10 cm, height 30 cm) to adapt for 10 min. Then, two withdrawal symptoms, paw tremors and head twitches, were assessed within 40 min. These withdrawal symptoms were selected based on reports of the most common rimonabant-precipitated cannabinoid withdrawal symptoms [56].

### 4.6. Data Analysis

Statistical analyses and graphical presentations of all experimental data were conducted with GraphPad Prism 9 (GraphPad Software, Inc., La Jolla, CA, USA). Data were expressed as mean ± SEM. Statistical comparisons between groups were performed by one-way or two-way analysis of variance (ANOVA) followed by the Bonferroni post hoc tests. A probability value (*p*) < 0.05 was considered to be statistically significant.

## 5. Conclusions

The present study demonstrated the tetrad effects, rewarding effect and physical dependence induced by three indazole-carboxamide SCs 5F-ADB, MDMB-4en-PINACA and ADB-4en-PINACA in mice. They all exhibited similar but stronger behavioral effects compared with Δ^9^-THC. Our present findings support the view that the three SCs have strong abuse potential and highlight their unpredictable effects with the potential for severe adverse reactions. The rank order of abuse potential for these compounds is 5F-ADB > MDMB-4en-PINACA > ADB-4en-PINACA > Δ^9^-THC. The result provides a foundation for further in-depth mechanism research in the future.

## Figures and Tables

**Table 1:** ED_50_ values and 95% confidence intervals (CIs) (mg/kg, i.p.) for Δ^9^-THC, 5F-ADB, MDMB-4en-PINACA and ADB-4en-PINACA in tetrad effects assays.

Drug	Suppressed Locomotion	Hypothermia	Analgesia	Catalepsy
	ED_50_ (95%CI) (mg/kg)	ED_50_ (95%CI) (mg/kg)	ED_50_ (95%CI) (mg/kg)	ED_50_ (95%CI) (mg/kg)
Δ^9^-THC	4.30 (3.07–6.98)	14.48 (10.05–23.21)	20.08 (15.76–25.84)	21.19 (15.27–45.27)
5F-ADB	0.03 (0.02–0.10)	0.07 (0.05–0.11)	0.04 (0.03–0.05)	0.03 (0.03–0.04)
MDMB-4en-PINACA	0.08 (0.06–0.21)	0.29 (0.21–0.80)	0.11 (0.09–0.13)	0.09 (0.02–0.14)
ADB-4en-PINACA	0.28 (0.20–0.46)	0.77 (0.68–0.86)	0.52 (0.40–0.63)	0.77 (0.59–1.41)

**Table 2:** Drug administration doses of rimonabant-precipitated withdrawal assay.

Drug	Administration Doses (mg/kg), i.p.				
	Day 1	Day 2	Day 3	Day 4	Day 5
Vehicle	0	0	0	0	0
Δ^9^-THC	1	3	10	30	100
5F-ADB	0.003	0.01	0.03	0.1	0.3
MDMB-4en-PINACA	0.01	0.03	0.1	0.3	1
ADB-4en-PINACA	0.03	0.1	0.3	1	3

## Data Availability

Data are contained within the article.
